# Understanding adaptive capacity and capacity to innovate in social–ecological systems: Applying a gender lens

**DOI:** 10.1007/s13280-016-0831-4

**Published:** 2016-11-22

**Authors:** Philippa J. Cohen, Sarah Lawless, Michelle Dyer, Miranda Morgan, Enly Saeni, Helen Teioli, Paula Kantor

**Affiliations:** 1 WorldFish, Jalan Batu Maung, Batu Maung, 11960 Bayan Lepas, Penang Malaysia; 2 Australian Research Council Centre of Excellence for Coral Reef Studies, James Cook University, Townsville, Australia; 3 Deakin University, Melbourne, Australia; 4 School of Anthropology, James Cook University, Townsville, QLD 4810 Australia; 5 WorldFish, Solomon Islands Office, P.O. Box 438, Honiara, Solomon Islands; 6 International Maize and Wheat Improvement Center, Texcoco, México

**Keywords:** Agriculture, Community, Development, Fisheries, Pacific, Resilience

## Abstract

Development policy increasingly focuses on building capacities to respond to change (adaptation), and to drive change (innovation). Few studies, however, focus specifically on the social and gender differentiation of capacities to adapt and innovate. We address this gap using a qualitative study in three communities in Solomon Islands; a developing country, where rural livelihoods and well-being are tightly tied to agriculture and fisheries. We find the five dimensions of capacity to adapt and to innovate (i.e. assets, flexibility, learning, social organisation, agency) to be mutually dependant. For example, limits to education, physical mobility and agency meant that women and youth, particularly, felt it was difficult to establish relations with external agencies to access technical support or new information important for innovating or adapting. Willingness to bear risk and to challenge social norms hindered both women’s and men’s capacity to innovate, albeit to differing degrees. Our findings are of value to those aspiring for equitable improvements to well-being within dynamic and diverse social–ecological systems.

## Introduction

The well-being of 700 million people globally is dependent on social–ecological systems via agriculture and fisheries. Well-being encompasses objective and subjective measures of a quality of life which in social–ecological systems are, by definition, linked to ecological processes (FAO 2003; Garcia and Cochrane 2005). Social–ecological systems are subject to high variability and change that impact upon human well-being (Walker et al. 2004). Many social–ecological systems in tropical developing countries have been the focus of emergency aid responses to help people cope with the impacts of severe shocks such as natural disasters or political unrest. Simultaneously, many of these systems are associated with chronic poverty and low levels of human well-being, and have been the focus of international development efforts. In many cases, however, emergency aid and development investments have failed to lead to long-lasting improvements to well-being, or to broader development outcomes for the most poor and marginalised (Meinzen-Dick et al. 2003).

There has been a global shift in development policy and practice to explicitly recognise that change, instability and uncertainty are inherent in social–ecological systems, and strongly influence people’s ability to derive benefits from natural resources and to realise improvements to well-being. Evidence, increasingly suggests that strategies that focus on building infrastructure or providing technical innovations, developed externally and delivered locally (to fishers and farmers, for example), are not realising lasting impacts, and/or do not bring benefit to the poor and marginalised (Slater and Tacchi 2004; Sumberg 2005). In response to this evidence, development practice has more recently become focused on building resilience and reducing vulnerability of communities and individuals within social–ecological systems (Walker et al. 2004; Lemos et al. 2007a; Brown and Westaway 2011). Adaptive capacity is a component of both resilience and vulnerability (Adger 2006). Resilience-building or vulnerability-reducing approaches identify the importance of recognising, protecting and strengthening inherent capacities of communities and individuals to deal with inevitable change, and also to drive change in a manner that will lead to wide-spread and sustainable improvements to well-being.

Adaptations are the actions of individuals, communities and governments undertaken for the purpose of improving or protecting well-being (Adger et al. 2005). Adaptation can be constrained or enabled by socio-institutional factors related to the physical, economic and social environment. Adaptive capacity refers to the conditions that enable people to anticipate and respond to change, and recover from and minimise the consequences of change (Adger and Vincent 2005). We use adaptive capacity to refer to women’s and men’s latent abilities to navigate inevitable change. We refer to capacity to innovate as the conditions that enable people to create and harness social or technical innovations. We use capacity to innovate to refer to inherent abilities of men and women to instigate favourable change. An innovation is an initiative, process or programme that changes existing routines, resource flows, and may be something entirely new or a novel recombination of established and new ideas (Moore and Westley 2011). Capacity to innovate can be viewed as one component of adaptive capacity (e.g. Eakin and Lemos 2006). In this paper, however, we treat capacity to innovate separately to examine people’s capacity to drive change, i.e. to transition or transform a system from its current state (Geels and Kemp 2007), as opposed to a response to an external change. In this way, we seek to understand what might constitute these inherent capacities to adapt and to innovate in the first instance.

Contemporary literature increasingly recognises that capacities to adapt and innovate are shaped by socio-institutional factors, including social identities and power relations, which include gender inequalities (Brown and Westaway 2011). Gender inequality has been widely acknowledged to adversely affect development outcomes and well-being at individual, household, community and national levels (United Nations 2010; World Bank 2012). Despite this insight, in many development efforts, “women continue to be underrepresented and underserved, and their contributions are not fully tapped” (Meinzen-Dick et al. 2014, p. 374). Of particular importance to our research focus, few studies have examined adaptive capacity and capacity to innovate in a manner that accounts for social, and particularly gendered, differences. As a result, many agricultural and natural resource management development and research interventions proceed in social–ecological systems oblivious to social and gender inequalities. This can reinforce existing inequitable power relations and the unequal distribution of benefits (Resurreccion and Elmhirst 2009; Meinzen-Dick et al. 2011b).

In this paper, we focus on *generic* capacities to adapt and to innovate, as opposed to understanding *specific* capacities to adapt to a pre-identified risk or particular hazard (e.g. increased extreme weather events due to climate change), or to innovate to address a pre-specified problem (sensu Lemos et al. 2007b; Tompkins et al. 2008). Adaptive capacity has been well described in abstract terms; however, scholars continue to be challenged to identify generic and practical determinants of adaptive capacity (Adger and Vincent 2005; Lemos et al. 2007a). To overcome this barrier, we examine our results through a framework that defines five dimensions of adaptive capacity: assets, flexibility, learning, social organisation and agency (McClanahan and Cinner 2012; Cinner et al. 2015). This framework allows analysts to identify obstacles and options for building capacities to adapt and to innovate. The five dimensions reflect constituents of adaptive capacity identified by other scholars. For example, Lemos et al. (2007a) summarise that scholars agree that adaptive capacity is built on availability of resources (captured here in ‘assets’), information and knowledge (captured here in ‘learning’) and institutions that enable change (here in ‘flexibility’ and ‘social organisation’). The framework we use also includes an ‘agency’ dimension, given that Brown and Westaway (2011) highlight that psychosocial factors have, to date, rarely been considered in analyses of adaptive capacity. For parallel frameworks, see Yohe and Tol (2002) and Eakin and Lemos (2006), and Smit and Wandel (2006).

The objective of this paper is to bring together nuanced understandings of social and gender differentiation with understandings of capacities to adapt and innovate. We present and analyse empirical data from a qualitative study examining how gender shapes capacities to adapt and innovate across three sites in Solomon Islands. Specifically, we look beyond quantifiable environmental or technological assets influencing capacities to adapt and innovate, to address two questions: (1) What socio-institutional factors shape capacity to adapt and to innovate in these contexts, and in what ways? And, (2) In what ways do social and gender norms shape these factors and thus influence differential capacity to adapt and innovate for women and men? In this paper, we begin by presenting the study area and methods. We then analyse and discuss the results in relation to the two questions, and draw insights for development interventions.

## Materials and methods

### Study area

**Fig. 1:**
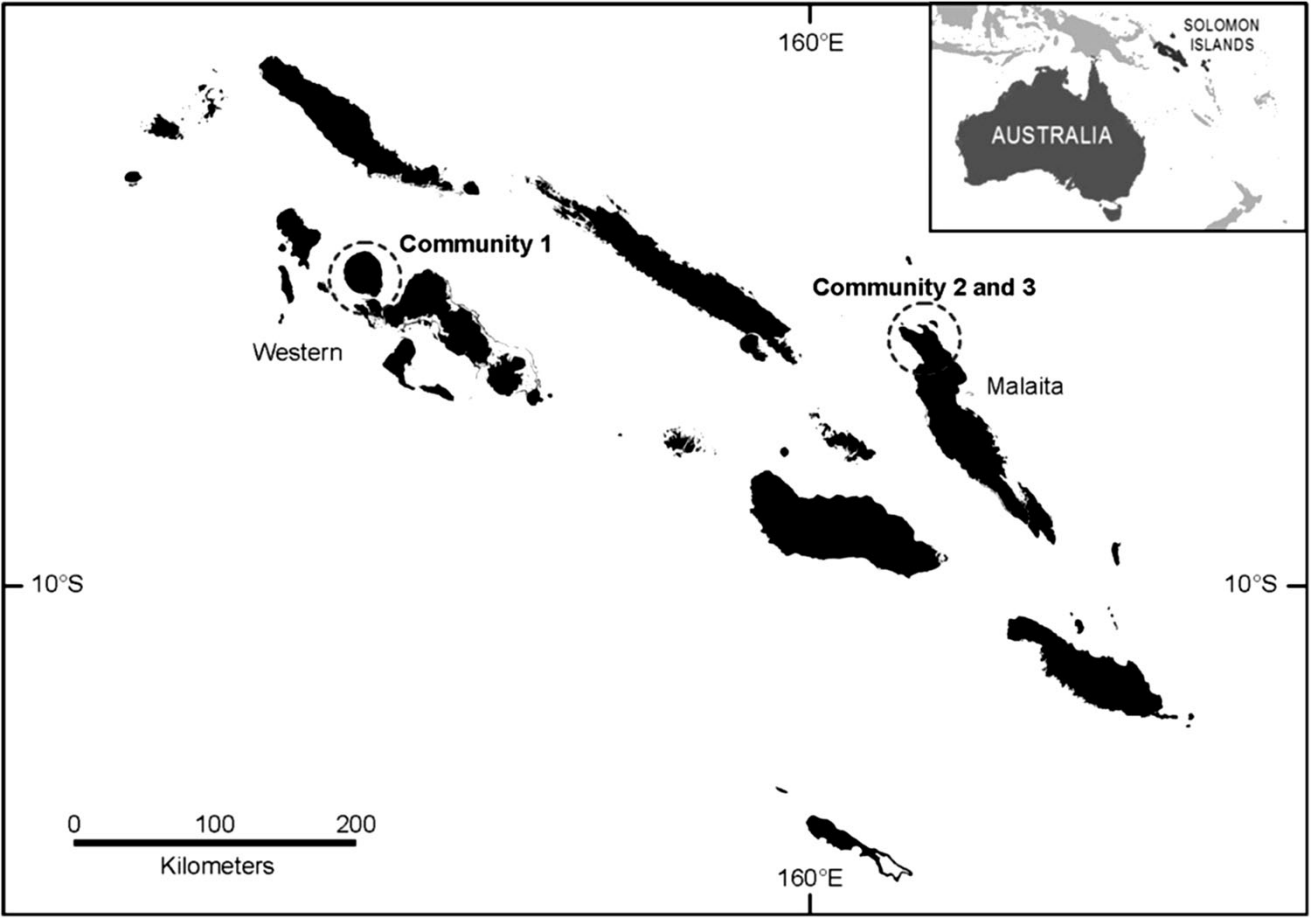
A map of Solomon Islands indicating the areas where the three study communities are located

Solomon Islands archipelago consists of nearly 1000 islands in the south-western Pacific Ocean (Fig. 1). The country is inhabited by around 600 000 people making it the third most populous Pacific Island country. Human population growth is high at 2.4 % per annum and the total population is projected to almost double by 2050 (UNESCAP 2009). Solomon Islands is considered to be of low levels of human development; ranking 157 out of 187 countries based on the United Nation’s Human Development Index (Malik 2014). Poverty in Solomon Islands is described as ‘poverty of opportunity’ as there are few opportunities for people to change or improve their living situations (Lightfoot and Ryan 2001).

In Solomon Islands, governance of land and coastal resources falls under the mandates of centralised and provincial government departments, but simultaneously governance is also heavily decentralised through customary land and sea tenure systems that are recognised in the national constitution (Lane 2006). The country is vulnerable to chronic stresses (related to poverty, remoteness and a poorly performing economy), and stochastic disturbances such as political instability and natural disasters such as earthquakes, cyclones and flooding. Over 80 % of the total population live in rural areas and rely predominantly on subsistence and small-scale agriculture and fisheries for food and income, with less than 20 % participating in salaried employment (Solomon Islands National Statistics Office 2009). Solomon Islands provides a useful case study because there is a substantial environment and development policy emphasis on promoting local innovation and adaptation—in part due to limitations in capacity of the central government. Lessons from this case will be of value for other analyses of social–ecological systems associated with agriculture and fisheries, developing country contexts and decentralised development efforts.

We undertook research in three rural and coastal communities: two communities in Malaita Province and one in Western Province (Fig. 1). These communities were selected because they were participating in an ongoing research programme^1^ (in which the authors were involved) and they (1) displayed a high reliance on agriculture and fisheries for food and livelihoods, (2) experienced resource decline or demise of livelihoods associated with agriculture or fisheries, and (3) had expressed an interest in improving the conditions of their social–ecological systems. Each community comprises between four and ten villages (Table 1), and we refer to these as single communities due to the geographical proximity and historical social alliances of the villages. Community names are not provided because of confidentiality arrangements.

**Table 1:** Size of communities in which research was conducted, completion rates of focus group respondents, number of focus groups, interviews and participants of each community, and education participation

	Western province	Malaita province	
	Community 1	Community 2	Community 3
No. villages	7	4	10
No. households	50+	72	68
No. FGD’s	8	8	8
FGD respondents (male:female:youth)	25:29:11	18:26:30	36:37:20
Interview respondents (male:female)	2:2	2:2	2:2
Primary education (% participation/completion)	100/61	87/39	91/72
Secondary education (% participation/completion)	54/0	27/1	52/1
No formal schooling (%)	0	13	9

Within these communities, people’s livelihoods were largely reliant on terrestrial (i.e. forest and agricultural plots) and/or aquatic resources (i.e. mangrove, reefs, sea, freshwater rivers and ponds). In initial community consultations, people had expressed concern about declining soil fertility and reductions in crop production, and reported decreased abundance of marine resources. All communities had access to basic health care, but more serious treatments required people to travel to regional hospitals. Access to clean water and sanitation was felt to be a problem in all communities. Access to markets required travel by foot, paddle canoe or motor boat and, respondents reported that remote geographical locations, rough seas, fuel expenses and poor road conditions made transporting goods to market difficult and inconsistent. In describing visions for the future during preliminary consultations, men and women expressed a desire to improve access to health services, sanitation and education, and improve community infrastructure, the management of natural resources and options to pursue profitable livelihoods. Respondents also discussed desired change in non-asset terms such as improving community governance and cohesion, and maintaining traditional values.

We collected data between September 2014 and September 2015. At that time, research programme activities were in their early stages. Activities included community consultation, preliminary scoping and agreement to research, the participatory development of a community action plan, some preliminary training and information sessions on fisheries management and organic farming techniques. Data collection described here represents a baseline of the social–ecological state rather than an assessment of programme activities.

### Data collection

We conducted focus group discussions (FGDs) separately with men, women and youth (Table 1). Youth were largely unmarried women and men of the ages of 16–24 who were active participants in agricultural and fisheries activities. Social norms which position men as public spokespeople meant that women and youth would be less likely to contribute to discussions in mixed groups, and so focus groups were separated to ensure that different perspectives were captured. FGDs were held with between five and twenty people who had volunteered their participation after a community meeting where we had discussed research objectives and processes. Discussants were people who resided locally and were engaged in agriculture or fishing. FGDs were held over numerous days, and rosters were developed to facilitate participation.

A total of eight FGDs were held in each community using four formats. The FGD (and interview described below) was adapted from GENNOVATE (Badstue et al. 2015); a global and comparative research initiative examining gender norms and agency in agriculture and natural resource management. Each FGD format was designed to examine a broad thematic area: (1) community and individual well-being, (2) social norms associated with household roles and livelihood activities (e.g. what it is to be a ‘good’ man or woman) and; (3) self- and collective-efficacy around strategic life decisions, particularly related to livelihoods. The final FGD format was designed specifically to gather youth perspectives and employed a combination of questions from the three formats described above. Questions were designed to explore social and gender differentiation of how individuals and communities were equipped to navigate and instigate change. We focussed particularly on agriculture and fisheries livelihoods, and explored in detail people’s perceptions of the gender dimensions to division of labour, decision-making within the household and broader expectations of moral behaviour.

We employed a semi-structured key informant interview to explore the innovations instigated by particular individuals. Semi-structured key informant interviews were conducted with women and men who were identified by community leaders as people who were ‘innovators’, and who had then agreed to be interviewed. Interview questions examined individual and contextual factors (including the influence of social and gender norms) that fostered or hindered the respondents’ learning, testing, uptake and adaptation of technical or social innovations related to their livelihood.

### Data analysis

FGDs and interviews were recorded digitally and in writing. All respondents provided prior verbal informed consent. FGDs and interviews were conducted in Solomon Islands Pijin and took between 40 min and 5 h. Interviews were then translated from Pijin into English using the digital and written recordings, and the English transcriptions were recorded in Word. Data were coded using qualitative data analysis software NVivo10. Preliminary coding was done using a coding structure developed from the themes addressed in the global GENNOVATE study. The coding structure consisted of theory-driven codes (related to the overarching themes of gender norms, agency, agriculture, natural resource management) and data-driven codes (based on sampling a sub-set of transcripts from three countries in the research programme, including Solomon Islands). Once data were coded, inductive reasoning was applied to examine emergent themes and subsequently these themes were organised into the five dimensions of the framework for further analysis.

## Results

### Assets

**Fig. 2:**
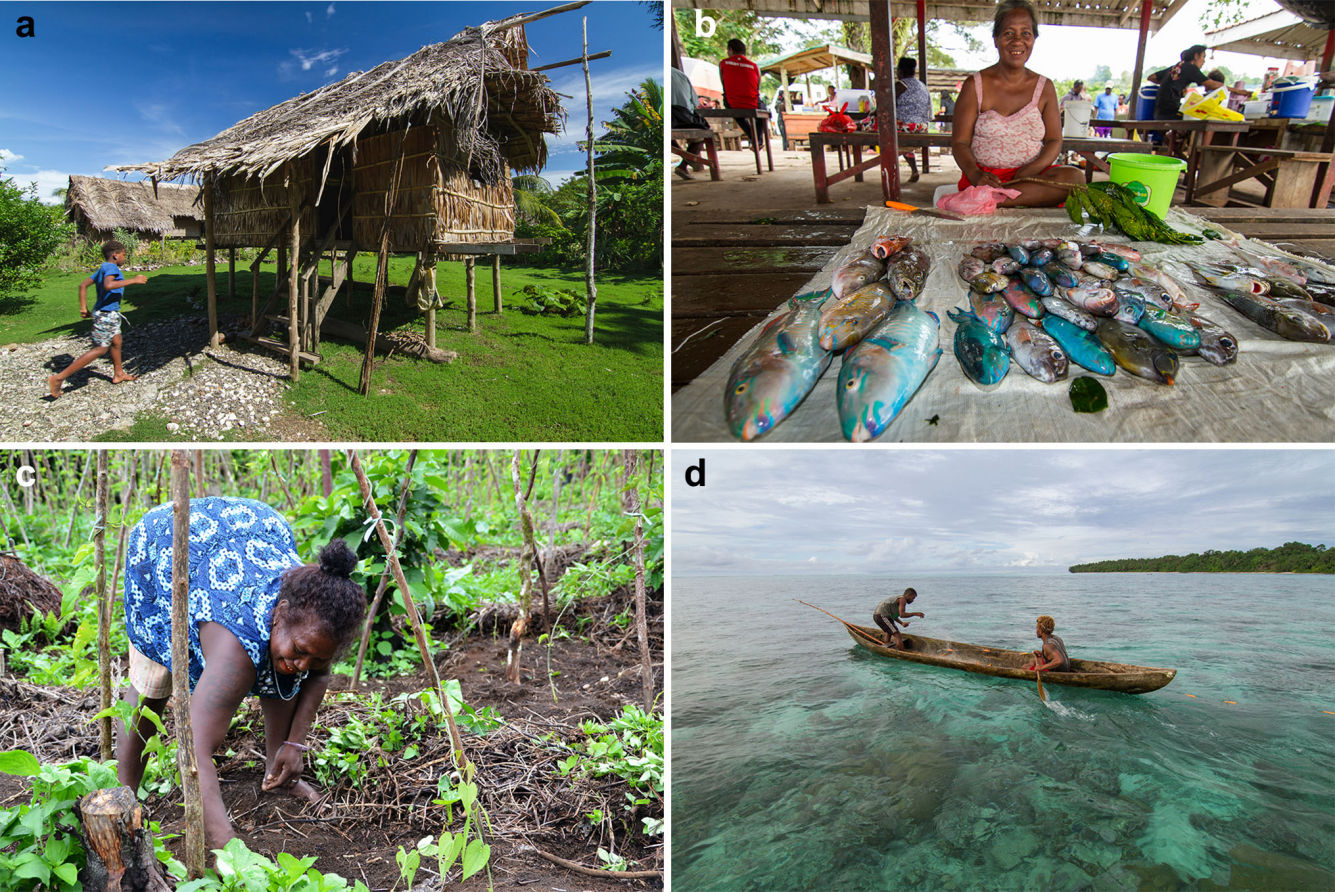
Panel figure depicting discussed elements of life and livelihoods in rural Solomon Islands, showing (clockwise); **a** a house in Malaita Province (photo by Filip Milovac), **b** a woman selling reef fish at the provincial capital market in Western Province (photo by Filip Milovac), **c** gardening in a small-scale agricultural plot (photo credit Jan van der Ploeg), **d** two men fishing with a net over reef from a dug-out canoe in Malaita Province a dug-out canoe used for subsistence and small-scale fishing (photo by Filip Milovac)

Most people in all three communities resided in sago palm-thatched houses, and households generally had basic equipment for farming and fishing (Fig. 2). The challenges that communities faced (as identified in early engagements with the research programme) were commonly associated with assets (e.g. land access and quality, health and transportation services). Some respondents expressed that a lack of money or tools inhibited their capacity to be innovative. For example, respondents reported being unable to access electricity, freezers or ice for chilling fresh produce which meant that they could not accumulate products or develop new products to take to market. Other respondents suggested that a lack of money in fact promoted innovation; one example provided was of a farmer who produced copra^2^ without access to conventional building tools or equipment, and had built his own copra dryer using local bush materials. In describing household well-being, respondents suggested there was no, or very little, difference between status of people and households within their communities. Yet, in general terms respondents did make some distinctions between people of ‘lower’ or ‘higher’ well-being, and a majority of these descriptors were assets (Table 2).

**Table 2:** A cumulative list from focus groups of characteristics of people or households of higher or lower well-being. The letter in brackets indicates the dimension(s) of adaptive capacity to which that characteristic relates (i.e. A = assets, L = learning, S = social organisation, Ag = agency, F = flexibility)

Lower well-being	Higher well-being
Live in leaf house (A) Couples that live with their parents (S) Need to borrow farming and fishing tools (A) Money is spent on alcohol (A) Limited household assets (A) Children do not attend school (A, L) Live in a community with poor sanitation and water supply (A) Do not work with other community members (S) Do not go to church (S) Live in a community with poor leadership (S) Live in a community where men and women engage in gossip (S) Live in a community in which men are lazy (S) Dependent on *wantoks* ^a^ (S) Poor knowledge of agricultural practices (L) Are not active in seeking income earning opportunities (L, Ag)	Live in permanent house with iron roofing (A) Own their own tools for fishing and farming (A) Money is spent on store bought foods (A) Access to marine and land resources (A) Large and productive agricultural plot (A) Hire labour to work in agricultural plot (A, S, F) Children attend school (A, L) Engage in paid employment (A, S, F) People are educated (L) People are creative and make use of their talents (L, Ag) Live in a community with a big permanent church (A, S) People who dress well (A) People who have a loving family (S) People who help and encourage others (S, L) Live in a community with a strong Church leader (S) People in a community work together (S)

^a^From the English “one talk”, refers to a person or group of people that share the same language, kinship group, geographical origins and common belief in mutual reciprocity (Nanau 2011)

Access to social, natural, physical and economic assets were socially and gender differentiated. The clearest distinctions in access to assets between people within communities were due to social norms, and were particularly evident for education (which we viewed as an asset), land and marine areas and cash (described further in ‘agency’). We found most respondents had accessed some level of primary education, and fewer people had secondary education (Table 1). Only female respondents reported having attained no formal education. Education completion varied between respondents and depended, largely, on their family’s ability to afford school fees. In community 1, a women’s ‘savings club’ associated with marketing of agricultural products was one innovation that had reportedly helped women save school fees for their own children. Higher levels of education correlated with greater access to other opportunities, for example, in community 3, women who had received more education said that it had allowed them greater exposure to paid employment or facilitated access to learning opportunities (further described in ‘learning’).

Given the high rates of reliance on agriculture and fisheries, access and use rights to land and marine areas were viewed as a foundation for the maintenance and improvement of well-being. Land and marine tenure operated through a system of patrilineal descent in communities 2 and 3, and matrilineal descent in community 1. All people in a community, regardless of origin, were given access to enough land for subsistence purposes. Men or women, however, who had migrated into communities due to marriage did not usually have ‘primary’ tenure rights, which meant they may not have had rights to make decisions about developments (e.g. establishing commercial enterprises) on land. In community 1, a mixture of women and men held primary rights to land, and theoretically had a voice in decision-making about natural resources use and management. In practice, however, men with primary rights often acted as spokespeople and negotiators in land matters.

### Flexibility

Flexibility was discussed in terms of moving up or down the well-being ‘ladder’ (i.e. a metaphor used in FGDs), livelihood mobility and physical mobility. Initiating improvements to household well-being was reported by some women to be a man’s responsibility; “*a man helps his family go up the ladder by putting a bush knife [for working in agricultural plots] in the hand of each of his family members and telling them to go and work*”. People believed that women needed to be committed to their husband’s plans in order to improve their household well-being. Most respondents reported that if women had their own money, her husband would have final say on how it was spent. In contrast, in community 1, women were reportedly leading improvements for their households due to their involvement in the communal savings club, and these women were often significant contributors to household income. Women reported that, *“men want to ask for help* [to improve their household’s well-being], *but they are ashamed. They are worried that others may talk about them negatively*”, and that these attitudes hindered men’s ability to drive improvements for their household.

Respondents described a general trend of increased reliance on the cash economy. Some women and men believed that life was harder compared to the past because “*everything depends on money. Life now is expensive*”. Whereas, some men believed that “*money makes everything easy*”. In all communities, increased access to cash by men was linked to increases in alcohol-related incidents, and women suggested that alcohol consumption hindered people’s ability to improve their well-being.

Growing engagement with the cash economy was also related to increased physical mobility, for men in seeking paid employment opportunities outside the village and for women in travelling to regional markets to sell fresh produce (Fig. 2). It was evident that social norms, and shifts in norms, influenced flexibility. Some women were restricted in their mobility and felt physically confined to the village, and as a result were limited in their livelihood options; one woman reported, “*some of us women only have a garden* [agricultural plot] *for our livelihoods*”. Such restrictions reduced women’s perceptions about their ability to engage with new ideas, whereas greater freedom to move outside the village related to greater agency, increased confidence and more opportunities to trial new practices.

Generally, discussions indicated that the gendered division of labour had become less rigid than in the past. For example, in community 2, women had become more involved in fishing, particularly net fishing, which was once an activity conducted only by men. In some cases, people felt that women now had heavier workloads due to taking on tasks previously performed only by men, in addition to their usual workload. Evidence suggested that, on occasion, some men engaged in household labour more than in the past when, according to custom, men were forbidden from cooking and washing women’s clothes; “*men before didn’t do all kinds of work… Men were the boss and were served by the women like a chief… But today, the men cook and they even do the washing*”. Whilst the partial relaxation of gender divisions in domestic roles was perceived to be favourable, other people retained the view that it challenged masculine status, for example, a woman should not be allowed to travel to market because her husband would be forced to undertake “*women’s work*” and that people “*…will say* ‘*she must be the boss of her husband*’”.

### Learning

Learning was discussed largely in terms of participating in training, seeking information, trialling new techniques and taking up new innovations associated with improving livelihoods; and as such, learning related strongly to capacity to innovate. Specific examples included social innovations such as resource management committees, the women’s savings club, and new technical innovations associated with fishing and farming methods, i.e. the use of modern fishing nets and lines, and organic farming. Given these changes were not responses to environmental or social shocks, we considered these to be innovations and illustrative of the application of capacity to innovate (i.e. rather than adaptations illustrating the application of adaptive capacity). Access to information, assessment of risks and social norms were cited as being influential on learning. People’s willingness to trial new practices or technology was influenced by the way in which they learned about them. Respondents discussed several examples where external organisations had delivered training and information. People reflected that the practical modes of information delivery or training (such as hands-on demonstrations, regular visits from trainers, along with verbal and practical encouragement and advice), rather than theoretical or hands-off modes of training, had more often led to people trialling and adopting new approaches.

Respondents felt they could actively seek-out information and support to guide innovation or adaptation through agricultural networks,^3^ extension agents,^4^ non-government organisations (NGOs), kin and broader social networks. Some people, however, felt they did not know what resources were available to them or how to access those resources. According to men, people of lower well-being (as defined in Table 2) tended to source information and support primarily from family or other people of similar well-being. In contrast, those considered of higher well-being (particularly related to education or social standing within the community) were able to seek-out support externally via staff from the government or from NGOs. Access to support and information was also gendered; men had more exposure to information and training than women. Women reported that access to new information was restricted by their lack of physical mobility and education, for example “*if I was able to read and write I would go and see those people* [holding formal positions] *in the* [government or NGO] *office, but I can’t read or write so it’s hard for me to go*”. Respondents also reported that the greater a women’s prior exposure to training and outside organisations, the more able, confident and willing she was to engage in further activities; “*before I just stayed in the village … people didn’t know who I was but now* [an outside organisation] *have chosen me to attend training … now I join most workshops that come into the community. That’s how I’ve changed*”.

Individuals and households were generally hesitant to learn through trialling new practices independently (i.e. without the support of an external agency). People felt that the risk or cost of delayed rewards, or no rewards at all, was too high to bear and they would face further hardship in the process of change. It was perceived that people’s willingness to adopt new practices would be higher with prior evidence of success, “*… people in the village want to see results first before they try new things*”. Women in particular expressed reluctance to trial new agricultural practices because they perceived there to be a risk that they would go hungry in the process of change, “*those who practise organic farming go hungry for some time until they start to reap the yields*”. Respondents also reported they relied on external organisations to initiate and support innovations. When people spoke about past experiences they suggested that once innovations were introduced by external agencies, there had been little adaptation or further innovation; “*we just follow what we were trained on*”. Some respondents reported that some people did trial new methods, but could be subject to taunting by other community members, for example “*… when a person does something new some people will mock them*”.

### Social organisation

Social organisation was discussed in terms of leadership, community governance structures, agricultural support groups and social networks for learning (described in ‘learning’). There was a sense that the onus of innovation and adaptation rested, somewhat, with those in formal leadership positions. Village chairmen, chiefs, elders and Church leaders were considered to be responsible for initiating changes, solving problems, resolving disputes and making other decisions within communities. There was evidence, however, that this reliance on leaders was shifting as all communities reported having recently formed village committees associated with the research programme, initially to “*look after the sea and land*” (see also results in ‘agency’). These committees included a broader representation than those previously described as ‘leaders’, and were established to help the community work together and to facilitate links with external organisations.

Respondents felt that the Church held an influential role in maintaining community cohesion and binding the community to work together to maintain well-being or to navigate change. Further, in community 3, small groups of the Church congregation provided help to families in their agricultural plots. Men reported being supported via access to agricultural networks and/or NGOs, whereas some women felt that women’s church groups were the only organisations that offered them support when they faced difficulties in their livelihood pursuits. One notable exception was the women’s savings club that had been instigated by an external agency in community 1, which had taught the women basic financial management skills and provided a village-based banking facility for women. Women reported that their involvement with the club had increased their access to financial capital, increased their participation in community and household decision-making and increased their confidence, particularly in their willingness to trial new innovations, including developing collaborative gardening schedules to increase crop yields and income.

### Agency

Agency was discussed in terms of people’s ability to make their own choices, or participate in household or community decisions that might influence their ability to cope with, or drive change to improve their well-being. Males were regarded as the head of the household as illustrated by male youth who reported, “*in our custom man must be a little bit on top*”, and female-headed households were rare. Older women who had been married longer were perceived to have more power and freedom to make decisions within the household compared to those women who were newly married. There was a general belief that a husband and wife should make joint decisions, In practice, however, men tended to have the final say in many household decisions and it was felt that a “*wife must obey her husband*”. Male and female respondents felt that men often made poor decisions concerning household money, and spent money on alcohol and things that were deemed unnecessary, “*… it is the men who ruin their wives money. They want to be the boss over their wives’ money …*”.

Men reported that, in the past, chiefs and other leaders made all the decisions in the community, and the *“people just followed what they said”*. Whereas, in more recent times, people had greater autonomy to make their own life decisions; “*…in the past only one or two leaders made all the life decisions for the people. But today everyone can make their own life decisions so we have more power nowadays compared to the past*”. Respondents suggested, however, that formal leaders were still responsible for making community-related decisions, indicating that, to differing degrees, traditional leadership structures still persisted. This change was, nevertheless, distinctly gendered with men remaining in positions of overall authority at community and household levels and within the Church hierarchy. Despite this, most women and men agreed that local changes in social organisation (such as women’s savings clubs) had increased women’s voice in household and community decision-making.

## Discussion

Instability and change in social–ecological systems can strongly influence human well-being. Our study adds to other empirical efforts that unpack adaptive capacity (Yohe and Tol 2002; Folke et al. 2003; Jones and Boyd 2011) and capacity to innovate (Rogers 2003) in dynamic social–ecological systems. The purpose of our study was not to quantify capacities or look narrowly at adaptations to particular shocks or pre-identified innovations. Our study examined five broad dimensions to build an understanding of the socio-institutional constitutes of adaptive capacity and capacity to innovate within rural communities. In particular, our study responds to the insight that the capacities of individuals, households and communities to adapt and innovate are influenced by social identities, relationships and norms (Brown and Westaway 2011). Whilst there is rich body of literature examining different processes and outcomes of social differentiation within rural development contexts, to date, there have been few examinations of how the constituents of adaptive capacity and capacity to innovate are shaped by social and gender norms. Our analysis highlights some areas and ways in which development interventions that seek to build adaptive and innovative capacities could be more sensitive to social and gender differences.

Traditionally, emergency aid and development interventions have tended to focus on just one dimension, i.e. the delivery of assets (e.g. financial assistance or technical provisions), as a means to ‘fix’ complex and diverse problems within socio-ecological systems (Folke et al. 2002; Degnbol et al. 2006). There are concerns about the efficacy of these reactive or ‘asset-only’ approaches in reducing vulnerability and bringing lasting improvements to well-being (Gilligan and Hoddinott 2007; Bermant 2008). Often asset-only approaches neglect to acknowledge other dimensions that may be enabling or inhibiting people to anticipate and respond to change (Adger and Vincent 2005). Access, control and ownership of financial, physical and productive assets enable people to create stable and productive lives, and play a role in shaping adaptive and innovative capacity (Meinzen-Dick et al. 2011a). This is often more complex in practice, as illustrated by our findings that both women and men were concerned that increased financial assets were not necessarily leading to improvements in household well-being, adaptive capacity or capacity to innovate—particularly where there were challenges faced in other dimensions.

Development interventions have paid insufficient attention to socially differentiated access and control over assets (Meinzen-Dick et al. 2011a; Quisumbing et al. 2014). In our study, respondents initially associated higher well-being with greater access to assets (Table 2). Deeper examination, however, highlighted that social disparities in access to assets were frequently related to strengths or weaknesses in other dimensions. For example, a women’s limited decision-making power regarding land and financial assets constrained her overall agency and flexibility to trial new livelihood opportunities. In Solomon Islands, ownership of land and coastal areas differs according to rules of inheritance and can be dynamic based on social exchanges (Hviding 1996; Foale and Macintyre 2000). Whilst women and men reported they had access to land and natural resources for subsistence purposes, access rights may not be sufficient to provide the freedom for innovation as social and gender norms place limitations upon who can make decisions about its use (Meinzen-Dick et al. 2011a). These findings offer insight to future analyses seeking to employ this framework of five dimensions, i.e. the interactions between dimensions are, arguably, as important as the differences found within the dimensions themselves.

In our study, we find both women’s and men’s abilities to maintain their well-being, and to adapt and to innovate, were indeed influenced, in part, by the availability and access to assets (e.g. education, equipment for farming and fishing, money, land and health and transportation services). By definition and unsurprisingly, the ability of people to maintain well-being is not built on assets alone. Learning and experimentation were influenced by access to information, and the ability and willingness to bear risk; these could in turn be influenced by the presence, absence or quality of relationships with external organisations. Features of social organisation, such as community governance structures, shaped both women’s and men’s ability to participate in community level decision-making, thereby influencing self- and collective-efficacy. Socio-institutional factors may differ in the way they promote or hinder the adaptive and innovative actions of different individuals (Narayan and Walton 2000; Klerkx et al. 2010). In sum, we found that social and gender norms shaped differences in women’s and men’s capacities, for example, in accessing support and information, participating in community governance and social organisation, and learning and experimenting.

People’s ability to act independently and make their choices freely (i.e. agency) is a crucial, but lesser studied, attribute of adaptive capacity (Brown and Westaway 2011). Both women and men in our cases articulated that decision-making has tended to rest with a few formal male community leaders, yet there were signs of shifts towards more devolved decision-making and the emergence of social innovations (e.g. committees and clubs) that promoted democratic processes and built individual agency. Women felt a level of autonomy in household decisions relating to agriculture and marketing. Decisions regarding physical and financial assets, however, including land use and commercial ventures, tended to be dominated by men (see also Quisumbing and Maluccio 2000). In addition to community decision-making, there were other signs that gender norms were changing. In the cases we examined, to a limited extent, traditional gendered divisions of labour were destabilising and this was in turn driving changes in social norms that allowed women and men greater livelihood flexibility. Our findings suggest that in some cases more livelihood opportunities may lead to a heavier labour burden, for women in particular (observed elsewhere, see Chant and Sweetman 2012). The insight that livelihood diversity might equate to a burden has been somewhat overlooked in adaptive capacity and resilience literature, which tends to emphasise the correlation between high livelihood diversity and high adaptive capacity (e.g. Cinner and Bodin 2010; Kotschy et al. 2015). This finding highlights that what may confer latent adaptive capacity (i.e., that which may serve well in the event of a social or environmental shock), does not necessarily equate to improved well-being in the current situation.

The ability of societies to act and respond collectively also influences their ability to deal with change or drive favourable change (Adger 2003). Community governance arrangements in many rural settings commonly feature a few powerful elites (almost always men) as leaders (Bennett 2002). In some circumstances ‘command and control’ leadership structures may perform well in initiating collective action, but are correlated with lower levels of individual agency (Cleaver 2007). We found emergent groups, such as the women’s savings club and women’s church groups, to be influential for increasing women’s self-efficacy, social and economic security, and greater prosperity for women and their families. The savings club that respondents discussed represented a social innovation that had continued to evolve independently since it was introduced. This success is in part attributable to the good social ‘fit’ of the innovation itself (Rogers 2003). Elsewhere evidence suggests that women’s social inclusion and increased agency in household and community decision-making can be built through such groups (Cornwall 2014). Our findings add weight to suggestions of others—that fostering these groups is a practical step to build innovative and adaptive capacities (Adger 2003).

Capacities to adapt and innovate are shaped by attributes of social organisation that enable or hinder people’s ability to draw on resources outside of their households or communities to cope with, or drive change (Pelling and High 2005; Rogers 2003). An individual’s capacity to access resources is strongly influenced by social norms built upon relationships of reciprocity and exchange (Adger 2003). In our case, gender imbalances in education, physical mobility, agency and social standing meant that men were more readily able to access and utilise new sources of information—a phenomenon observed elsewhere (Meinzen-Dick et al. 2011b). Certain people, women and youth in particular, were less able to establish relations with external agencies to access new information. As a result their capacities to innovate and adapt were restricted. Successfully promoting women’s capacities to adapt and innovate will require deliberate and informed “*investment in [*women’s*] capacity to respond creatively to emerging opportunities, more trust in their knowledge, and sensitive, supportive accompaniment*” (Cornwall 2014). Development assistance must account for the differences in people’s ability to access and utilise training, knowledge and resources so as not to exacerbate existing inequalities, or inadvertently perpetuate the exclusion of already vulnerable or marginalised groups (Pelling and High 2005).

Capacity to innovate tends to be higher in people who are risk-takers or have reasonable assurances that they will benefit from their efforts in experimenting (Berdegué 2005) or adopting innovations (Rogers 2003). We found that risk aversion was common to both women and men but that, in line with findings of others, women seemed particularly risk averse (Fothergill 1996; Eckel and Grossman 2008). There is a clear link between learning and agency, where evidence of success can inspire and motivate further innovation as it raises people’s beliefs that they can create desired effects through their actions (Bandura 1998). Importantly, evidence of failure can produce the opposite outcome. External organisation, such as government or NGOs, can play an important role of ‘innovation broker’ (sensu Klerkx and Gildemacher 2012) to initiate innovations, promote their uptake and help carry some of the risk or cost of experimentation.

Individual self-efficacy and self-perceptions of competence are indicators of agency (Brown and Westaway 2011). Many development programmes see low self- or collective-efficacy as an entry point to focus on building local empowerment. Our findings emphasise that low self-efficacy and collective-efficacy stem from, or are reinforced by, deficiencies in other dimensions. This was apparent, for example, through women’s and men’s perception that their ability to drive improvements in individual and household well-being were constrained by limits in assets, learning and flexibility. Therefore, efforts that focus on local empowerment, must also acknowledge and account for deficiencies in other dimensions. Our study has focused on individual and community adaptive capacity and capacity to innovate; however, efforts to build these capacities must also consider where capacity deficiencies are reinforced by socio-institutional structures beyond the local scale (Adger et al. 2005; Smit and Wandel 2006). Programmes and policies that promote decentralisation and ‘local empowerment’, i.e. the intention to shift the responsibility for innovation and adaptation to citizens, will not facilitate improvements to well-being if they are naïve to the risks and costs of experimentation and adaptation that people will face.

## Conclusion

Understanding social and gender differences of capacities to adapt and innovate is imperative to achieve more socially inclusive development processes, greater equitability in outcomes and more sustained improvements to well-being. In this study, we sought to differentiate adaptive capacity from capacity to innovate but concede that, depending on the context, the conditions that enable adaptation may be very similar to those that enable innovation (Klerkx et al. 2010). In certain circumstances an innovation may well be part of an adaptation strategy (e.g. Eakin and Lemos 2006). Further, we have examined adaptive capacity and capacity to innovate at just one point in time. Adaptations and innovations may well occur *within* the context we have described using a framework of five dimension. But in other circumstances, adaptations and innovations may in fact transform the very rules that currently govern the system (Geels and Kemp 2007; Moore and Westley 2011). The framework of five dimensions we have employed has been built from largely Western conceptions of the constituents of adaptive capacity, but importantly does not necessarily align with local epistemologies of what constitutes adaptive capacity and capacity to innovate. There is fertile ground for further research to understand differing conceptions of these capacities, and builds understandings of how dimensions of generic adaptive capacity and capacity to innovate play out through time in the face of specific hazards or shocks, and as particular innovations unfold and spread.

We found capacities to adapt and innovate to be shaped by a range of related socio-institutional factors, in particular, pressure to conform to social norms, willingness to bear risks, need for evidence, power structures embedded in social relationships and organisation and access to information. In practice there may be trade-offs, synergies and conflicts between generic adaptive capacity and adaptive capacity for specific risks or hazards (Lemos et al. 2007b), and these are likely to spread costs and benefits unevenly within societies (Brown and Westaway 2011). Whilst our study has demonstrated the importance of examining within a local scale, efforts to understand and build capacities must also recognise the cross-scale influence of actors, policies and contexts in hindering or facilitating adaptation and innovation, and broader well-being (Adger et al. 2005; Geels and Kemp 2007).

Three important insights are evident from our study for development practice. First, local social relations and norms were strongly explanatory in understanding differences in people’s potential to cope with, or drive change. Second, there is a legitimate role for external agencies to carry risk associated with innovation that might be too costly or risky for local innovators to overcome. And finally, our study has highlighted that all five dimensions of capacity to adapt and innovate, and interactions between dimensions, can vary substantially between people based on gender or other social determinants. The application of a gender lens in this study has been particularly insightful for understanding how interventions might promote (or undermine) social inclusion and equitable improvements to people’s capacities to adapt and innovate. Implementing development or research initiatives in a manner sensitive to these differences will be less likely to exacerbate existing inequality, and more likely to promote change that will help people navigate and drive change in dynamic social–ecological systems.

## Footnotes

1 The CGIAR Research Program on Aquatic Agricultural Systems is a research on development programme seeking new approaches to realise improvements to human well-being within aquatic agricultural systems.

2 The dried flesh of coconut from which oil is extracted.

3 Group of locally run agricultural organisations offering support to farmers in northern Malaita region.

4 Government representative employed to assist people in rural areas.

## References

[CR1] Adger N (2003). Social capital, collective action, and adaptation to climate change. Economic Geography.

[CR2] Adger N (2006). Vulnerability. Global Environmental Change.

[CR3] Adger N, Vincent K (2005). Uncertainty in adaptive capacity. Comptes Rendus Geoscience.

[CR4] Adger NW, Arnell WN, Tompkins EL (2005). Successful adaptation to climate change across scales. Global Environmental Change-Human and Policy Dimensions.

[CR5] Badstue, L., P. Kantor, G. Prain, J. Ashby, and P. Petesch. 2015. Innovation and development through transformation of gender norms in agriculture and natural resource management: A global comparative research initiative.

[CR6] Bandura A, Adair JG, Belanger D, Dion KL (1998). Personal and collective efficacy in human adaptation and change. Advances in psychological science: Vol. 1. Personal, social and cultural aspects.

[CR7] Bennett, J. 2002. Roots of conflict in Solomon Islands though much is taken, much abides: Legacies of tradition and colonialism. *State, society and governance in Melanesia, ANU, working paper series*.

[CR8] Berdegué, J. 2005. *Pro-poor innovation systems: Background paper.* Washington, DC: IFAD.

[CR9] Bermant, L.S. 2008. *Intrahousehold asset dynamics and its effect on the intergenerational transmission of poverty*. *A select annotated bibliography and literature review,* ed. Overseas Development Institute. London: Overseas Development Institute.

[CR10] Brown K, Westaway E (2011). Agency, capacity, and resilience to environmental change: Lessons from human development, well-being, and disasters. Annual Review of Environment and Resources.

[CR11] Chant S, Sweetman C (2012). Fixing women or fixing the world? ‘Smart economic’, efficiency approaches, and gender equality in development. Gender and Development.

[CR12] Cinner JE, Bodin Ö (2010). Livelihood diversification in tropical coastal communities: A network-based approach to analyzing ‘livelihood landscapes’. PLoS ONE.

[CR13] Cinner J, Huchery C, Hicks CC, Daw TM, Marshall N, Wamukota A, Allison EH (2015). Changes in adaptive capacity of Kenyan fishing communities. Nature Climate Change.

[CR14] Cleaver F (2007). Understanding agency in collective action. Journal of Human Development.

[CR15] Cornwall, A. 2014. Women’s empowerment: What works and why? *WIDER Working Paper Series 2014/14.* World Institute for Development Economics Research (UNU-WIDER).

[CR16] Degnbol P, Gislason H, Hanna S, Jentoft S, Nielsen J, Sverdrup-Jensen S, Wilson D (2006). Painting the floor with a hammer: Technical fixes in fisheries management. Marine Policy.

[CR17] Eakin H, Lemos M (2006). Adaptation and the state: Latin America and the challenge of capacity-building under globalization. Global Environmental Change.

[CR18] Eckel C, Grossman P, Plott C, Smith V (2008). Men, women and risk aversion: Experimental evidence. Handbook of experimental economic results.

[CR19] FAO. 2003. The ecosystem approach to fisheries: Issues, terminology, principles. *Institutional Foundations, implementation and outlook FAO fisheries technical paper 443*. Rome: FAO

[CR20] Folke C, Carpenter S, Elmqvist T, Gunderson L, Holling CS, Walker B (2002). Resilience and sustainable development: Building adaptive capacity in a world of transformations. Ambio.

[CR21] Folke C, Colding J, Berkes F, Berkes F, Folke C, Colding J (2003). Synthesis: Building resilience and adaptive capacity in socio-ecological systems. Navigating social–ecological systems: Building resilience for complexity and change.

[CR22] Fothergill A (1996). Gender, risk and disaster. International Journal of Mass Emergencies and Disasters.

[CR23] Garcia SM, Cochrane KL (2005). Ecosystem approach to fisheries: a review of implementation guidelines. ICES Journal of Marine Science.

[CR24] Geels FW, Kemp R (2007). Dynamics in socio-technical systems: Typology of change processes and contrasting case studies. Technology in Society.

[CR25] Gilligan D, Hoddinott J (2007). Is there persistence in the impact of emergency food aid? Evidence on consumption, food security, and assets in rural Ethiopia. American Journal of Agricultural Economics.

[CR26] Hviding E (1996). Guardians of Marovo Lagoon: Practice, place and politics in maritime Melanesia, Pacific Islands Monograph Series.

[CR27] Jones L, Boyd E (2011). Exploring social barriers to adaptation: Insights from Western Nepal. Global Environmental Change.

[CR28] Klerkx L, Aarts N, Leeuwis C (2010). Adaptive management in agricultural innovation systems: The interactions between innovation networks and their environment. Agricultural Systems.

[CR29] Klerkx L, Gildemacher P (2012). The Role of Innovation Brokers in Agricultural Innovation Systems.

[CR30] Kotschy K, Biggs R, Daw T, Folke C, West P (2015). Principles for building resilience. Sustaining ecosystem services in social-ecological systems.

[CR31] Lane MB (2006). Towards integrated coastal management in Solomon Islands: Identifying strategic issues for governance reform. Ocean and Coastal Management.

[CR32] Lemos MC, Boyd E, Tompkins E, Osbahr H, Liverman D (2007). Developing adaptation and adapting development. Ecology & Society.

[CR33] Lemos MC, Agrawal A, Eakin H, Nelson DR, Engle NL, Johns O, Asrar GR, Hurrell JW (2007). Building adaptive capacity to climate change in less developed countries. Climate science for serving society; research, modeling and prediction priorities.

[CR34] Lightfoot, C., T. Ryan, J. and Quitazol. 2001. *Poverty: Is it in an issue in the Pacific?* Manila: Asian Development Bank.

[CR35] Foale SJ, Macintyre M (2000). Dynamic and flexible aspects of land and marine tenure at West Nggela: Implications for marine resource management. Oceania.

[CR36] Malik K (2014). Human development report 2014. Sustaining human progress: Reducing vulnerabilities and building resilience.

[CR37] McClanahan TR, Cinner J (2012). Adapting to a changing environment: Confronting the consequences of climate change.

[CR38] Meinzen-Dick, R., M. Adato, L. Haddad, and P. Hazell. 2003. *Impacts of agricultural research on poverty: Findings of an integrated economic and social analysis*. Washington: International Food Policy Research Institute.

[CR39] Meinzen-Dick, R., N. Johnson, A.R. Quisumbing, J. Njuki, J. Berhman, D. Rubin, A. Peterman, and E.Waithanji. 2011a. Gender, assets, and agricultural development programs: A conceptual framework. *CAPRi Working Paper*. Washington: International Food Policy Research Institute.

[CR40] Meinzen-Dick, R., A.R. Quisumbing, and J.A. Behrman. 2014. A system that delivers: Integrating gender into agricultural research, development and extension. In *Gender in agriculture: Closing the knowledge gap*, ed. A.R. Quisumbing, R. Meinzen-Dick, T.L. Raney, A. Croppenstedt, J. Berhman, and A. Peterman, pp. 373–391. Rome: Food and Agriculture Organization of the United Nations.

[CR41] Meinzen-Dick, R., A.R, Quisumbing, J. Berhman, P. Biermayr-Jenzano, V. Wilde, M. Noordeloos, and N. Beintema. 2011b. *Engendering agricultural research, development and extension* Vol. 176. Washington, DC: International Food Policy Research Institute.

[CR42] Moore M, Westley F (2011). Surmountable chasms: networks and social innovation for resilient systems. Ecology & Society.

[CR43] Narayan, D., and M. Walton. 2000. Changing gender relations in the household. In *Voices of the poor: Can anyone hear us*? New York: World Bank Publications.

[CR44] Nanau G (2011). The wantok system as a socio-economic and political network in Melanesia. The Journal of Multicultural Society.

[CR45] Pelling M, High C (2005). Understanding adaptation: What can social capital offer assessments of adaptive capacity?. Global Environmental Change.

[CR46] Quisumbing, A.R., and J.A. Maluccio. 2000. Intra-household allocation and gender relations: new empirical evidence from four developing countries. *FCND Discussion Paper* (Vol. 84). Washington DC: International Food Policy Research Institute.

[CR47] Quisumbing AR, Meinzen-Dick R, Raney TL, Croppenstedt A, Berhman J, Peterman A, Quisumbing AR, Meinzen-Dick R, Raney TL, Croppenstedt A, Berhman J, Peterman A (2014). Closing the knowledge gap on gender in agriculture. Gender in Agriculture, Closing the Knowledge Gap.

[CR48] Resurreccion, B.J., and R. Elmhirst. 2009. Gender, environment and natural resource management: New dimensions, new debates. In *Gender and natural resource management: Livelihoods, mobility and interventions,* pp. 3–20. UK: Earthscan.

[CR49] Rogers EM (2003). Diffusion of innovations.

[CR50] Slater D, Tacchi J (2004). Research ICT innovations for poverty reduction.

[CR51] Smit B, Wandel J (2006). Adaptation, adaptive capacity and vulnerability. Global Environmental Change.

[CR52] Solomon Islands National Statistics Office. 2009. Report of Economic Activity and Labour Force. In *Solomon Islands populations & housing census*. Honiara: Solomon Islands Government.

[CR53] Sumberg J (2005). Systems of innovation theory and the changing architecture of agricultural research in Africa. Food Policy.

[CR54] Tompkins E, Lemos M, Boyd E (2008). A less disastrous disaster: Managing response to climate-driven hazards in the Cayman Islands and NE Brazil. Global Environmental Change.

[CR55] UNESCAP (2009). Statistical yearbook for Asia and the Pacific 2008.

[CR56] United Nations. 2010. *The millenium development goals report 2010*, New York.

[CR57] Walker B, Holling CS, Carpenter SR, Kinzig A (2004). Resilience, adaptability and transformability in social–ecological systems. Ecology and Society.

[CR58] World Bank. 2012. *World development report 2012: Gender equality and development*. New York: World Bank.

[CR59] Yohe G, Tol R (2002). Indicators for social and economic coping capacity moving toward a working definition of adaptive capacity. Global Environmental Change.

